# Assessment of Poor Home Management Practice of Diarrhea and Associated Factors among Caregivers of Under-Five Years Children in Urban and Rural Residents of Doba Woreda, Ethiopia: Comparative Cross-Sectional Study

**DOI:** 10.1155/2019/8345245

**Published:** 2019-06-02

**Authors:** Waktole Kebede Fufa, Gebretsadik Berhe Gebremedhin, Gebremedhin Berhe Gebregergs, Taklu Marama Mokonnon

**Affiliations:** ^1^Zonal Health Office, West Hararghe Zone, Chiro, Ethiopia; ^2^Department of Epidemiology, College of Health Sciences, Mekelle University, Mekelle, Ethiopia; ^3^Department of Midwifery, College of Health Sciences and Medicine, Wolaita Sodo University, Wolaita Sodo, Ethiopia

## Abstract

**Background:**

Diarrhea is the first cause of illness and the second cause of death in under-five children. Home interventions can prevent 57% of mortality related to diarrhea. However, malpractices were common and the reason for this underutilization was unclear. Thus, this study was aimed at assessing poor home management practice of Diarrhea and associated factors among caregivers of under-five years children in urban and rural residents.

**Methods:**

The community-based comparative cross-sectional study was conducted in Doba woreda, Ethiopia, from February 25 to March 15, 2017. Multistage cluster sampling technique was used to study 559 caregivers. An interviewer administered pretested structured questionnaire was used to collect data. Collected data were entered into Epi Info version 3.5.1 and exported to statistical package for social sciences (SPSS) version 20.0 for analysis. The binary logistic regression model was used. In bivariate analysis p-value<0.25 was taken into multivariable analysis. Adjusted odds ratios with their corresponding 95% of CI were used to report results with a significance level of p-value<0.05.

**Result:**

184 urban and 375 rural caregivers were included in the study. Poor home management practice was 55.8% of urban and 85.6% of rural residents. Knowledge level (AOR=2.7(CI[1.3, 6.5]) and AOR=13.4(CI[5.3, 34.0]) and difficulty in preparing oral rehydration salt (AOR=4.0CI[1.4, 11.0]) and AOR=2.4(1.3, 5.3)) were associated factors for both urban and rural residents, respectively. Caregivers of male index child (AOR=2.3(1.2, 4.7)) and age of the caregivers (AOR=0.26(0.09, 0.8)) were associated with poor home practice for urban residents. In rural residents, inaccessibility to zinc supplementation (AOR=2.4(1.2, 5.0)) was among associated factors.

**Conclusion:**

Poor home management practice of diarrhea was high in both urban and rural residents. It was higher in rural compared to urban residents. Poor practice was associated with knowledge level, age of the caregivers, sex of the index child, and accessibility of zinc. Health education and community mobilization on home management of diarrhea are important to increase awareness and improve practice level.

## 1. Background

Diarrhea is the unusual frequent passage of three or more loose or watery stools in the 24-hour period. It is caused by many types of microorganism and other factors and common in areas where poor access to safe water and consumption of contaminated food are prevalent and practices of personal hygiene are poor [1–4].

Drug therapy is unnecessary in most diarrheal cases and even contraindicated or dangerous, because the majority of diarrheal cases in children's are viral in origin and most of diarrheal cases can be managed by mothers at home without drug [2]. Whatever the cause or classification, mostly diarrhea results in losses of water and electrolytes (sodium and potassium) and is complicated by metabolic disorder and sometimes death [3, 5].

Globally, diarrhea is the second cause of mortality and the most common cause of illness in childhood and it is accountable for 1.5 billion cases and 2 million deaths per year [5, 6]. Diarrhea is more widespread in developing countries. African children experience average of five episodes of diarrhea per year and 15% of the time was spent with children with diarrhea in most prevalent area. In Africa, 800,000 of children lose their life annually, which accounts for 25% of all childhood death [7, 8]. In Ethiopia, diarrhea prevalence ranges from 11% to 37%. Ethiopia was ranked the fifth worldwide in perspective of total child death and around 73,700 children die each year due to diarrhea which accounts for 20% of all child death [9–11]. Death due to diarrhea is mostly associated with loss of water and electrolytes [3].

The role of the mother is important in health promotion, diarrhea prevention, and management of the sick child. Caregivers awareness and practice on fluid intake and child feeding during diarrheal episode are important [10]. Mothers are the key caregivers, who determine and decide the type of food and fluids given to the child. The overall management of diarrhea depends on mother's decision. Therefore, their level of knowledge and practice on diarrhea are critically important [12].

The usually recommended management of diarrhea is the use of oral rehydration therapy [13]. Rice water, yogurt, soup, salt sugar solution, and clean water are also recommended home based fluids. Low osmolarity oral rehydration salt (ORS) and zinc are also included as components of home management of diarrhea. Timely administrations of oral rehydration therapy [13] and zinc tablets have proved to be of less cost and efficient as principal management to reduce deaths from diarrheal disease [3, 6, 12, 14, 15].

Even though there was progress in advanced diagnostic methods, improved management, and increased utilization from health facility, diarrhea continued to be the main cause of morbidity and the second causes of mortality in under-five children. Diarrhea prevention and control efforts alone were less effective in reducing child mortality and should be complemented with good home management practice [12, 16, 17].

Globally 50% of children with diarrhea did not visit any health facility, 69.7% of them were managed at home with home available fluids. However, practices are inappropriate. Home management of diarrhea was the first option in rural mothers, due to inaccessibility of health facility [18]. In Africa, harmful practices such as food restriction (within the range of 30–60%) and fluid restriction (within ranges of 11%-80%) were common. This malpractice negatively affects child health and development [19].

The declined users of ORS and also incorrect preparation were the main problem [18]. Knowledge of mother of sugar salt solution (SSS) was lower (68.2%) than that of ORS and its actual preparation and use were very low (7%)[17]. Even in areas where ORS utilization is high, there is increasing concern that incorrect preparation and practices are inappropriate [20]. The reason for ORS underutilization was unclear [21]. In developing countries about 46% of the children were given less than the usual amount of food and few mothers gave yogurt and rice water for their children for the management of diarrhea [17, 22]. This research is aimed at assessing the magnitude of poor home management practices of diarrhea and associated factors among caregivers of under-five children in urban and rural residents of Doba woreda, Ethiopia.

## 2. Methods and Materials

### 2.1. Study Design

Comparative cross-sectional study was conducted among caregivers of under-five children in urban and rural residents.

### 2.2. Ethics and Consent to Participate

Ethical approval was obtained from Mekelle University Collage of Health Sciences, ethical review board (IRB). Before commencement of this study, official permission of letters was obtained from the Oromia Regional Health Bureau to the Zonal Health Department and from Zonal Health Department to Woreda Health Office. The participants were informed of the purpose of the study and privacy during the interview. Informed written consent was obtained from each study participant prior to interviewing. They were also made aware that they have the full right to participate or not to participate in the study and to withdraw anytime during the interview. Confidentiality also was maintained through the coding of questionnaire and made anonymously.

### 2.3. Study Area and Period

The study was conducted in Doba “*woreda*” (third-level administrative divisions of Ethiopia), Western Hararghe, Oromia region, Eastern Ethiopia. There are fourteen “woredas” in West Hararghe zone and Doba district is one of the fourteen districts. The woreda has the total population of 174,813 with forty rural and two urban “*Kebeles*” [“neighborhood” is the smallest administrative unit of Ethiopia]. About 37,194 estimated households are available. Under-one children were 5629 (3.22%) and under-five children were estimated to be 28,721 (16.43%) of the population. Doba town has access to electricity, piped water, road access, digital telephone, postal service, and banking. Health service coverage of the woreda was 98%. The study in woreda has health service system like six public health centers, forty health professionals, and five private clinics.

Severe acute malnutrition, pneumonia, and diarrhea were the three top five causes of morbidity in under-five children. Acute watery diarrhea outbreak also repeatedly occurs in woreda. Nonbloody diarrhea is the first leading cause of morbidity [23]. The study was conducted from February 25 to March 15, 2017.

### 2.4. Study Population and Participants

Study populations are all caregivers of 6-59-month children in randomly selected kebeles. Participants of the study are all caregivers of 6-59-month children who were selected and included in the study from each selected “Got” (the subdivision of Kebele with total members of 25-30 households).

### 2.5. Eligibility Criteria

All caregivers of 6-59-month children are residents in the specific place for greater than 6 months, available at home during data collection period, and those above 15 years old were included in the study. But those who were critically ill and cannot hear or see were excluded.

### 2.6. Sample Size Determination

To determine sample size, two population proportion formulas of unmatched sample size were used. Assumption of 95% CI, 80% power of the study, 17% urban and 34% proportion of rural residents (2:1 urban rural ratio) [24], design effect 2, and 5% of nonrespondents were considered. The calculated sample size was 264. Considering design effect of 2, n=2*∗*264, n=*528*, by adding 5% of nonrespondents, the final estimated sample size was* 554* (185 from urban and 369 from rural) participants.

### 2.7. Sampling Technique

Multistage cluster sampling was used. Kebeles and “*Gots*” (small division of kebele) in the woreda were selected by simple random sampling (lottery method) from their respective sampling frame (urban and rural). Two urban Kebeles and 25% of the rural Kebeles were selected. Accordingly, ten rural and two urban Kebeles were sampled. At the second stage, 30 Gots were sampled from rural and 15 Gots were sampled from urban selected kebeles. From each Got, 9-18 caregivers had been interviewed. Totally, 184 from urban and 375 from rural residents were included in the study. If at least one under-five child was available at home with the primary caregiver, she was invited to participate in an interview. If primary caregiver was not available, the household was revisited one time or more.

### 2.8. Outcome Variable

Home management practice of caregivers on childhood diarrhea is categorized as a poor home management practice or good home management practice. Components of home management of childhood were fluid intake (mainly ORS), zinc supplementation, and food intake during diarrheal episode. These components were known by three rules [4, 25].

### 2.9. Data Collection Tools and Techniques

Data was collected using adopted structured questionnaire [26]. The questionnaire consists of close-ended questions. The questionnaire was subdivided into five sections: sociodemographic characteristics, fluid intake, feeding practices, use of zinc supplementation, and other elements of home management practices. Six data collectors and two supervisors were recruited to conduct this research. Diploma holders and above were selected as the data collector. Health extension workers and health workers [27] were excluded from data collection. Data was collected through an interviewer administered questionnaire. The interview was performed in the house-to-house way at the community level.

### 2.10. Data Management and Processing

Variables were coded and transformed as necessary. Missing value was checked and treated in the same way. Data was entered and cleaned into Epi Info version 3.5.1. Then, it was exported to statistical package for social sciences (SPSS) for analysis.

### 2.11. Data Analysis and Presentation

Categorical variables such as place of residence, gender, level of education, sex of child, marital status, religion, and occupation were computed and presented in tables and bar charts. Age, household size, and days that the child had diarrhea were reported with their mean and standard deviation. Hosmer-Lemeshow goodness of fit test was performed to check whether the binary logistic regression model fits with data or not. The model fitness of both urban and rural residents was 0.79 and 0.45 p-value, respectively. None of the independent variables had multicollinearity in this study.

To identify the associated factors, binary logistic regressions model was used. Variables with p-value <0.25 at the bivariate logistic regression were taken for multivariable analysis. Adjusted odds ratio with 95% CI was used to report results with a significance level of p-value<0.05.

### 2.12. Data Quality Assurance

The selected data collectors and supervisors were trained for two days on content, study process, study tool administration, ethical considerations, and data entry.

The research tool was pretested on 5% of the sample size from the nonsampled study population (28 caregivers). Questions clarity to respondents and applicability with local context were assessed and adjustments were made as necessary. The interview questionnaire was translated into Afaan Oromo.

Two diploma nurse supervisors were assigned. Each day, the questionnaires were screened carefully for completeness, clarity, correctness, and consistency. The meeting was conducted with data collectors every two days to discuss the study progress and make adjustments regarding the data collection process as necessary. Data was cleaned and analyzed carefully with Epi Info.

### 2.13. Operational Definition

 
*Poor Home Management Practice of Diarrhea.* Poor practice was described by an aggregate score of below three important components of home management of diarrhea (ORS intake, continued or increased feeding, and zinc supplements)[4, 25].
 
*Good Home Management Practice of Diarrhea.* Participants answered three rules and above of home management of diarrhea, such as increased fluid intake (commonly ORS solution), continued or increased feeding, and zinc supplementations [4, 25].
 
*Practices.* Practice refers to the act (response) to diarrhea by the caregiver as the home management of diarrhea attributed through the questionnaires and verbal response [2].
 
*Good Knowledge on Home Management of Diarrhea.* Those caregivers were able to answer seven and above (above the mean) of the knowledge related 13 questions measured as good knowledge of home management of diarrhea [10].
 
*Poor Knowledge on Home Management of Diarrhea.* Caregivers were able to answer below the mean (seven) of the knowledge related 13 questions measured as poor knowledge [10].

## 3. Result

### 3.1. Sociodemographic Characteristics

A total of 559 caregivers (184 residents and 375 rural residents) were recruited into the study, with a response rate of 100%. The mean age of the caregivers was 27.0±6.0 (SD) years and ranges from 17 to 50 years. The average number of people in one household was 4.7±2.0 (SD). The mean age of the index child included in the study was 26.0±13.4 (SD) months.

Caregivers who cannot read and write were 22 (12%) of the urban residents and nearly half 199 (53%) in the rural residents. Seventy two (39.1%) of urban residents and 21 (5.7%) of rural residents attend secondary education. Those who were governmental employed were 33 (19.6%) of the urban and 3 (0.8%) of the rural residents. One hundred and thirty-seven (74.5%) of urban and 331 (88.3%) of rural residents were those of the followers of Muslim religion. Five hundred and forty-six (97.7%) of the caregivers were biological mothers. The number of under-five children in one household with two children was 259(45.4%) (Table 1).

### 3.2. Diarrhea Prevalence

The two-week period prevalence of diarrhea was 26 (14%) at 95% CI of 9.8% and 19.9% in urban residents and 97 (26%) at 95% CI of 21.7% and 30.5% in rural residents. The total diarrhea period prevalence was 123 (22%). Sixty-two (11%) of interviews caregivers reported that their child did not have any episode of diarrhea in last one year. One hundred and sixty-three (88.9%) of the urban residents and 334 (89.3%) of the rural residents reported that their child had an episode of diarrhea in last one year.

**Figure 1:**
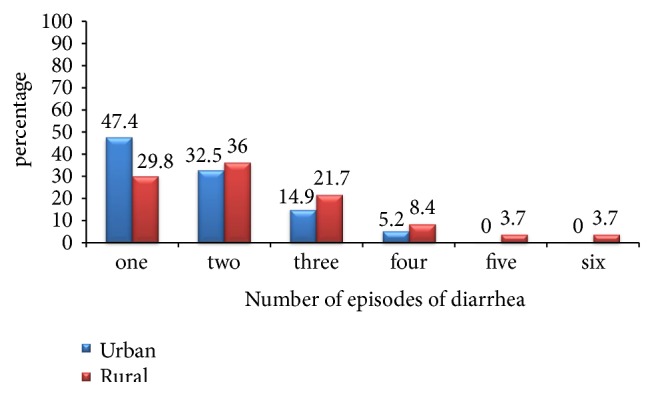
Number of episodes of diarrhea per one year among under-five children in urban and rural resident of Doba woreda, Ethiopia, 2017.

The average number of episodes of diarrhea in last one year was 2.1±1.2 (SD) and ranges from 1 to 6 times. The sum of one and two episodes of diarrhea per one year accounts for 80% in urban areas and 65.8% in rural areas. However, the sum of both five and six episodes of diarrhea accounts for 7.4% in rural residents and none of the urban residents had five and more episodes of diarrhea per year. The average duration of one episode of diarrhea was 3.5±1.7 (SD) days with a range of 1-15 days (Figure 1).

### 3.3. Knowledge and Practice on Home Management of Diarrhea

**Figure 2:**
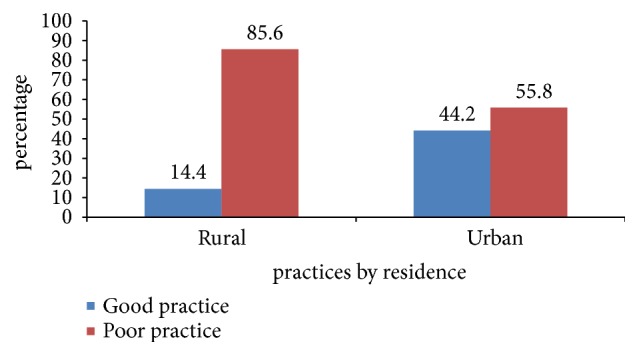
Magnitude of home management practice of diarrhea among caregivers of under-five children in urban and rural resident of Doba woreda, Ethiopia.

A majority of 149 (81%) of urban residents and nearly one third 145(38.7%) of rural residents had good knowledge of home management of diarrhea. Out of 497 children with diarrhea, 377 (75.9%) at 95% CI (71.8%, 79.5%) had a poor home management practice. The magnitude of poor home management practice was 91 (55.8%) at 95% CI (47.8%, 63.5%) in urban and 286 (85.6%) at 95% CI (81.3%, 89.1%) in rural residents (Figure 2).

### 3.4. Fluid Intake during Diarrhea

Ninety-nine percent had heard about ORS as home management of diarrhea. Four hundred and seventy-four (84.8%) caregivers knew as one liter of water is needed to dissolve ORS. Awareness of the salt-sugar solution was 173 (30.9%). About 168 (30.1%) caregivers withheld at least one type of fluid during a diarrheal episode. During home visits, 74 (13.2%) of respondents had ORS at their house (Table 2).

### 3.5. Feeding of the Child during a Diarrheal Episode

About 454 (81.2%) respondents believed that breast feeding is important during a diarrheal episode. In total, 32 (5.7%) caregivers withheld any type of food (Table 3).

### 3.6. Zinc Supplementation

One hundred and fifty-two (82.6%) urban and 141 (37%) rural residents had heard about zinc medicine. The correct awareness of duration of zinc treatment (11-15 days) was 103 (56%) in urban and 39 (10.4%) in rural residents. Nearly half 266 (47.6%) of the caregivers did not know the importance of zinc for diarrhea treatment (Table 4).

Of total respondents 293 (52.4%) who had heard about zinc, most of the 139 (92%) urban and 115 (81.6%) rural residents had heard from the local health workers. Caregivers who heard from radio were 8 (5.3%) in urban and 22 (15.6%) in rural residents. Five urban (3.3%) and 4 (2.8%) rural residents had heard about zinc from their neighbors.

### 3.7. Place of Care of Sick Child

**Figure 3:**
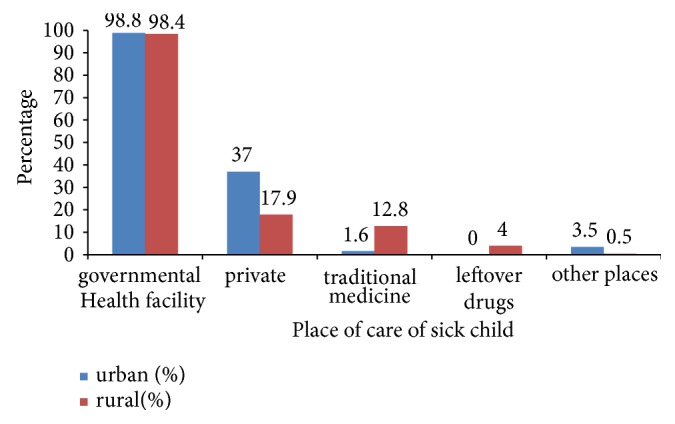
Place of care for sick child among caregivers of under-five children in urban and rural resident of Doba woreda, Ethiopia.

The most utilized in place for the care of sick child was governmental health facility 370 (98.4%) in rural and 181 (98.8%) in urban residents (Figure 3).

### 3.8. Home Management Practices

Decreased fluid intake 186 (55.7%) and decreased food intake 181 (54.2) were the same for rural settings. SSS utilization was 24 (14.7%) in urban and 71 (21.3%) in rural residents. Caregivers, who gave rice water for their sick child, reached11 (6.7%) in urban residents and 66 (19.8%) in rural residents. Those caregivers who gave yoghurt were 15 (9.2%) in urban residents and 51 (15.3%) in rural residents during a diarrheal episode. Home management of diarrhea differs among rural and urban residences (Table 5).

### 3.9. The Relationship between Residence and Selected Health Related Indicators

Home management practice of diarrhea, knowledge level of the caregivers on home management of diarrhea, history of diarrhea in last two weeks, household size, and place of delivery of index child had significant association with residence (Table 6).

### 3.10. Factors Associated with Home Management Practices of Diarrhea

The predictors of poor home management practices of diarrhea had some similarities and differences among rural and urban residents (Table 7).

Poor knowledge and difficulty in preparing ORS were associated factors of poor home management practice for both urban and rural caregivers. The odds ratio of poor home management practice among participants who had poor knowledge was thirteen [AOR= 13.4, 95% CI (5.3, 34.0)] and three [AOR=2.7, 95% CI (1.3, 6.5)] times higher than their comparator in rural and urban residents, respectively. The odds ratio of poor home management practice among caregivers who did not easily prepare ORS was two [AOR= 2.4, 95% CI (1.3, 5.3)] and four [AOR=4.0, 95% CI (1.4, 11.0)] times higher than their comparator in rural and urban residents, respectively, shown in Table 7.

In rural residents, the difficulty in getting zinc was 2 times more likely to have poor home management practices [AOR=2.4, 95% CI (1.2, 5.0)]. In urban residents, being the mother of male index child was 2.3 times more likely to have poor home management practices than being the mother of female index child [AOR=2.3, 95% CI (1.2, 4.7)]. In urban residents, caregivers with age of 26-35 years was 74% times less likely to have poor home management practices compared to caregivers with age of 36 years and above [AOR=0.26, 95% CI (0.09, 0.8)], shown in Table 7.

## 4. Discussion

The magnitude of poor home management practices of childhood diarrhea among rural residents was 1.8 times higher than urban residents. Poor knowledge and difficulty in preparing ORS were associated with poor home management practice of diarrhea in both urban and rural residents. Age of the caregiver and the sex of the index child in urban resident and accessibility of zinc in rural residents had significant association with poor home management practice of diarrhea.

The higher magnitude of poor home management practice in rural residents compared to urban residents was consistent with community based study done in Mareka district [2]. It might be related to awareness and access to health information of urban residents. This study also revealed that most of the urban (76.4%) caregivers had formal education as compared to rural (40%) ones. But the finding was in contrary with study conducted in Kenya [28]. This might be due to practice in rural area being obligatory where there is no access to modern health care treatment facility and urban residents seek medical care from health facility and did not practice these recommended interventions at home.

In terms of factors that were associated with home managements of diarrhea, finding of this study was inconsistent with study conducted in Mareka district [2] and rural Nigeria [17]. The inconsistence might be related to former studies that only used sociodemographic predictors and did not include other important variables such as awareness of salt sugar solution, yogurt, and accessibility of components of home management of diarrhea that were included in the current study.

In this study, 85.6% of rural and 55.8% of urban caregivers had poor home management practice of childhood diarrhea. The urban poor home management practice of diarrhea in this study was similar with study conducted in Ethiopia [10]. Poor home management practice of diarrhea was higher compared to study conducted in Mareka district (in rural (60%) and in urban (10%)) with their respective residence [2]. This might be related to difference in educational status and other socioeconomic and cultural difference.

In this study, knowledge level of urban caregivers was 2 times higher than rural caregiver. The odds ratio of having a poor home management practice was 13 times and 3 times higher in caregivers with poor knowledge as compared to those with good knowledge in urban and rural residents, respectively.

This study was consistent with study conducted in Finote Selam [10] and Mareka District [2]. It might be due to mothers' poor knowledge of the cause of diarrhea and component of home management of diarrhea that might limit them from taking timely and appropriate action for their sick child.

Level of awareness and utilization of zinc in this study was better than that of Kenya, Bankura, southern Nigeria, and India. In Nigeria, 4/771 of children were provided by zinc [28]. In Bankura only 2.9% of mothers heard about the zinc medicine [29]. No caregivers heard about zinc tablet in southern region of rural India and Nigeria [16, 20]. The higher awareness in this study might be associated with health policy of Ethiopia that the Federal Ministry of Health (FMoH) includes zinc as essential drug that should be available at local health facility and prescribed free of charge at health post level.

In this study, in urban residents, caregivers with age of 26-35 years were 74% times less likely to have poor home management practices of diarrhea compared to caregivers with age of 36 years and above. It was consistent with study in Finote Selam where older age caregivers (age of 36-45 years) were more likely to have poor home management practice compared to youngsters (age of 15-25 years) due to the current educational accessibility for young caregivers, but older caregivers were not more accessed to formal education [10].

In this study, sex of the index child was associated with level of practice in urban residents. Caregivers of male index child were 2 times more likely to have poor home management practices when compared to caregivers of female children. This finding was inconsistent with study conducted in Mareka district [2] and Arba Minch [30]. It also contrasts with prior expectation of sex preference in different culture and social value of males compared to females.

### 4.1. Strength and Limitation of Study

The strength of the study was that a similar study was not conducted in the study locality which gives clues to the level of home management practice of diarrhea and associated factors. Important predictor variables in this study were not included in former study that enriches the knowledge of home management practices of diarrhea.

Limitation of the study was that measurement of home management practice of diarrhea was based on the caregivers response that may not indicate the real practice of caregivers. There might be also a limited degree of recall bias due to the fact that caregivers had to remember the practice in last one year.

## 5. Conclusion

The magnitude of poor home management practice was high in both rural and urban residents. Moreover, the poor home management practice was higher in rural residents as compared to urban one. Commonly, poor knowledge level of caregivers of components of home management of diarrhea and difficulty in preparing ORS were statistically associated with poor home management practice in both urban and rural residents. Distinctly, availability and accessibility of zinc were significantly associated with poor home management practice in rural caregivers, whereas age of the caregivers and being caregivers of male index child were associated with poor home management practice in urban residents.

A majority of both urban and rural residents had low awareness and underutilization on rice water, yogurt, and salt sugar solution. Yoghurt and rice water were utilized more in rural residents than in urban residents.

## Figures and Tables

**Table 1:** Socio-demographic characteristics of caregivers of under-five children's in urban and rural resident of Doba woreda, Ethiopia, 2017.

Variables	Category	Residence		Total (n=559)
		Urban (n =184)	Rural (n=375)	
Age of the caregiver	15-25	80(43.5)	116(30.9)	196(35.06)
	26-35	79(42.9)	212(56.5)	291(52.0)
	>36	25(13.6.)	47(12.7)	72(12.4)

Religion	Muslim	137(74.5)	331(88.3)	468(83.7)
	Orthodox	33 (17.9)	41 (10.9	749 (13.3)
	Others*∗*	14(7.6)	3(0.8)	17(3.0)

Ethnicity	Oromo	168 (91.3)	353 (94.1)	521(93.2)
	Amhara	13(7.1)	21(5.6)	34 (6.1)
	Others*∗∗*	3(1.6)	1(0.3)	4(0.7)

Educational status	Can't read and write	22(12.0)	199(53)	221(39.5)
	Read and write	3(1.6)	23(6.1)	26(4.7)
	Primary	6 4(34.8)	130(34.7)	194(34.7)
	Secondary	72(39.1)	21(5.7)	93(16.6)
	Tertiary (college)	23(12.5)	2(0.5)	25(4.5)

Occupation	Housewife	89(48.4)	136(36.3)	225(40.3)
	Farmer	12(6.5)	186(49.6)	198(35.4)
	Merchant	32(17.4)	24(6.4)	56(10.0)
	Governmental employs	36(19.6)	3(.8)	39(7.0)
	Others *∗∗∗*	15(8.1)	26(6.9)	41(7.3)

Marital status	Married	174(94.6)	344(91.7)	518(92.7)
	Single	0	1(0.3)	1(0.3)
	Divorced	8(4.3)	21(5.6)	29(5.0)
	Widowed	2(1.1)	9(2.4)	11(2.0)

Relationship caregiver with child	Biological mother	181(98.4)	365(97.3)	546(97.7)
	Grand mother	2(1.1)	4(1.1)	6(1.1)
	Others*∗∗∗∗*	1(0.5)	6(1.6)	7(1.2)

Number of under-five children	1	117(63.6)	183(48.8)	300(53.7)
	2	66(35.9)	188(50.1)	254(45.4)
	3	1(0.5)	4(1.1)	5(0.9)

Place of delivery	Health facility	147(79.9)	188(50.2)	335(59.9)
	Home	37(20.1)	185(49.3)	222(39.7)
	Other places*∗∗∗∗∗*	0(.0)	2(0.5)	2(0.4)

Sex of child	Male	100(54.3)	182(48.5)	282(50.4)
	Female	84(45.7)	193(51.5)	277(49.6)

Age of the child in months	6-11	27(14.7)	37(9.9)	64(11.4)
	12-23	48(26.1)	113(30.1)	161(28.8)
	24-35	46(25.0)	112(29.9)	158(28.3)
	36-47	35(19.1)	56(14.9)	91(16.3)
	48-59	28(15.1)	57(15.2)	85(15.2)

Birth order of the index child	First	78(42.4)	101(26.9)	179(32.1)
	Second	57(31.0)	88(23.5)	145(25.9)
	Third	16(8.7)	44(11.7)	60(10.7)
	Fourth and above	33(17.9)	142(37.9)	175(31.3)

*∗*= Protestant, Catholic and Waqefata, *∗∗*=Gurage and Somali, *∗∗∗*= private work, Day laborer, Jobless, *∗∗∗∗*=father, relatives, *∗∗∗∗∗*= on the way to the health facility

**Table 2:** Awareness on fluid intake among caregivers of under-five children's in urban and rural resident of Doba woreda, Ethiopia.

Variables	Category	Urban(n=184)	Rural(n=375)	(n=559)
		No. (%)	No. (%)	No. (%)
Heard about ORS*∗*	Yes	184(100)	370(98.7)	554(99.1)
	No	0	5(1.3)	5(0.9)

Composition of ORS*∗*	Salt and sugar	111(60.3)	166(44.6)	277(49.6)
	Salt, sugar and mineral	53(28.8)	77(21.5)	130(23.9)
	Don't know/don't answer	20(10.9)	127(33.9)	147(26.5)

Amount of water used to dissolve ORS*∗*	0.5 liter	3(1.6)	15(4)	18(3.2)
	1 liter	172(93.5)	302(80.5)	474(84.8)
	2 liter and above	8(4.4)	36(9.6)	44(7.9)
	Don't know/don't answer	1(.5)	22(5.9)	23(4.1)

ORS available at home?	Yes	31(16.8)	43(11.5)	74(13.2)
	No	153(83.2)	332(88.5)	485(86.8)

Time to discard dissolved ORS	Until finished	6(3.3)	33(8.8)	39(7.0)
	<23Hours	29(15.8)	102(27.2)	131(23.5)
	At 24 hours	148(80.4)	188(50.1)	336(60.1)
	>25hours	0	11(2.9)	11(2.0)
	Don't know/don't answer	1(.5)	41(11)	42(7.5)

Had heard SSS *∗*	Yes	85(46.2)	88(23.5)	173(30.9)
	No	99(53.8)	287(76.5)	386(69.1)

Know how to prepare SSS	yes	61(33.2)	69(18.4)	130(23.3)

Prepared SSS previously	yes	34(18.5)	56(14.9)	90(16.1)

Withheld any type of fluid	Yes	48(26.1)	120(32.0)	168(30.1)
	No	136(73.9)	255(68.0)	391(69.9)

SSS*∗* “salt-sugar solution”, ORS*∗* “Oral rehydration salt”

**Table 3:** Knowledge and attitude of feeding practice among caregivers of under-five children's in urban and rural resident of Doba woreda, Ethiopia.

*Variables*	Category	Residence		Total (n=559)
		Urban(n=184)	Rural(n=375)	
		No. (%)	No. (%)	No. (%)
Feeding aggravate diarrhea?	No	172(93.5)	343(91.5)	515(92.2)
	Yes	12(6.5)	32(8.5)	44(7.8)

Breast feeding is important during diarrhea?	Yes	166(90.2)	288(76.8)	454(81.2)
	No	15(8.2)	74(19.7)	89(15.9)
	Don't know	3(1.6)	13(3.5)	16(2.9)

Do you Withhold any type of food?	Yes	8(4.3)	24(6.4)	32(5.7)
	No	176(95.7)	351(93.6)	527(94.3)

Did you hear of yogurt as home management of diarrhea?	Yes	44(23.9)	81(21.6)	125(22.2)
	No	140(76.1)	293(78.4)	433(77.8)

Yogurt is used as home management for diarrhea?	Yes	39(21.2)	74(19.7)	113(20.2)
	No	145(78.8)	301(80.3)	446(79.8)

What do you feel about the importance of yogurt?	Good	44(23.9)	77(20.5)	121(21.6)
	Not good	0(.0)	3(.8)	3(.5)
	Nothing	0(.0)	2(.5)	2(.4)
	Don't know	140(76.1)	293(78.2)	433(77.5)

Did you hear about Rice water?	Yes	58(31.5)	154(41.1)	212(37.9)
	No	126(68.5)	221(58.9)	347(62.1)

What do you feel about the importance of rice water?	Good	55(29.9)	150(40.1)	205(36.7)
	Not good	3(1.6)	0(.0)	3(.4)
	Nothing	0(.0)	2(.5)	2(.4)
	Don't know	126(68.5)	223(59.4)	349(62.5)

**Table 4:** Zinc supplementation for home management of diarrhea among caregivers of under-five children's in urban and rural resident of Doba woreda, Ethiopia.

Variables	Category	Residence		Total (n=559)
		Urban(n=184)	Rural(n=375)	
		No. (%)	No. (%)	No. (%)
Heard about Zinc?	Yes	152(82.6)	141(37.6)	293(52.4)
	No	32(17.4)	234(62.4)	266(47.6)

Duration to give zinc?	Until diarrhea stops	24(13.0)	42(11.2)	66(11.8)
	<10 days	19(10.3)	42(11.2)	61(10.9)
	10-14 days	103(56.0)	39(10.4)	142(25.4)
	≥15 days	1(.5)	5(1.3)	6(1.1)
	Don't know/don't answer	37(20.2)	247(65.9)	284(50.8)

Importance of zinc?	To shorten Duration	130(70.7)	115(30.7)	245(43.9)
	To stop diarrhea	14(9.6)	17(6.5)	31(8.5)
	Don't know/don't answer	32(19.7)	234(62.8)	266(47.6)

**Table 5:** Home management practice of diarrhea among caregivers of under-five children's in urban and rural resident of Doba woreda, Ethiopia.

Variables	Category	Urban(n=163)	Rural(n=334)	Total=497
		No. (%)	No. (%)	No. (%)
How much fluid did give since diarrhea starts?	More than usual	94(57.7)	50(15.0)	144(29.0)
	Less than usual	39(23.9)	186(55.7)	225(45.3)
	Same to usual	29(17.8)	92(27.5)	121(24.3)
	Nothing	1(.6)	6(1.8)	7(1.4)

Is ORS*∗* had given for child previously?	Yes	157(96.3)	289(86.5)	446(89.7)
	No	6(3.7)	45(13.5)	51(10.3)

Is SSS*∗* had given to child previously?	Yes	24(14.7)	71(21.3)	95(19.9)
	No	139(85.3)	263(78.7)	402(80.1)

Amount of food given since diarrhea?	More than usual	70(49.9)	34(10.2)	104(20.9)
	Less than usual	47(28.8)	181(54.2)	228(45.9)
	Same to usual	46(28.2)	113(33.8)	159(32.0)
	Nothing	0	6(1.8)	6(1.2)

Did you give yogurt since diarrhea starts?	Yes	15(9.2)	51(15.3)	66(13.3)
	No	148(90.8)	283(84.7)	431(86.7)

Did you provide rice water for sick child?	Yes	11(6.7)	66(19.8)	77(15.5)
	No	152(93.3)	268(80.2)	420(84.5)

Did you provide zinc for sick child?	Yes	93(56.2)	102(28.6)	195(39.2)
	No	70(43.8)	232(71.4)	302(60.8)

Amount of food given during convalescent period?	More than usual	121(74.2)	160(47.9)	281(56.5)
	Less than usual	3(1.9)	40(12.0)	43(8.7)
	Same to usual	39(23.9)	134(40.1)	173(34.8)

SSS*∗* “salt sugar solution”, ORS*∗* “Oral rehydration salt)

**Table 6:** An association between residence and selected health related factors among caregivers of under-five children's Doba woreda, Oromia, Ethiopia.

Variables	Rural	Urban	COR (95% CI)	P value
*Home management practice*				
Good	48	72	*0.212(0.14, 0.33)*	*0.001*
Poor	286	91	1.0	
*Level of knowledge*				
Poor	230	35	6.7*5(4.43, 10.03)*	0.001
Good	145	149	1.0	
*Two week history of diarrhea*				
No	275	158	*0.46(0.29,0.75)*	*0.002*
Yes	97	26	1.0	
*Educational status*				
No/Informal education*∗*	222	25	*9.2(5.7,14.7)*	*0.001*
Formal education*∗∗*	153	159	1.0	
*Household size*				
Less than five	185	131	*0.39(0.27,0.58)*	0.001
Greater or equal to five	190	53	1.0	
*Place of delivery*				
Home	185	37	3.96(2.9,5.9)	0.001
Health facility	188	147	1.0	
*Awareness on SSS∗∗∗?*				
No	287	99	2.8(1.9, 4.1)	0.00
Yes	88	85	1.0	
*Awareness on yogurt?*				
No	294	141	1.1(0.7, 1.68)	0.64
Yes	81	43	1.0	
*Awareness on Zinc?*				
No	234	32	7.8(5.1, 12)	*0.01*
Yes	141	152	1.0	

COR= Crude odds ratio, CI= confidence Interval, “1.0” indicates reference group, *∗*=can't read and write and informal education, *∗∗*= primary, secondary, tertiary, SSS*∗∗∗* “salt sugar solution”

**Table 7:** Associated factors of poor home management practice of diarrhea among caregivers of under-five children in urban and rural resident of Doba Woreda, Ethiopia.

Variable	Home management practice in rural				Home management practice in Urban			
	Poor=286	Good=48	COR (95%CI)	AOR (95%CI)	Poor=91	Good=72	COR (95%CI)	AOR (95%CI)
*Age category*								
16-25 years	117(82.4)	25(17.6)	0.7(0.28,1.7)		39(57.4)	29(42.6)	0.45(0.16,1.3)	0.39(0.13,1.3)
26-35years	123(88.5)	16(11.5)	1.2(0.45,3.0)		34(47.9)	37(52.1)	*0.30(0.11, 0.8)∗*	0.26(0.09,0.8)*∗*
>36years	46(86.8)	7(13.2)	1.0		18(75)	6(25)	1.0	1.0

*Educational status*								
No/Informal education*∗∗∗*	176(89.8)	20(10.2)	2.24(1.2,4.2)*∗*	1.6(0.8,3.2)	17(73.9)	6(26.1)	2.5(0.9,6.8)*∗∗*	1.1(0.33,3.7)
Formal education *∗∗∗∗*	110(79.7)	28(20.3)	1.0	1.0	74(52.9)	66(47.1)	1.0	1.0

*Occupation*								
Housewife(farmer)	247(86.1)	40(13.9)	1.3(0.5,2.9)		54(62.1)	33(37.9)	1.7(0.9,3.2)*∗∗*	0.88(0.4,1.87)
Other *∗∗∗∗∗*	39(83.0)	8(17.0)	1.0		37(48.7)	39(51.3)	1,0	1.0

*Household size*								
Less than five	139(84.8)	25(15.2)	0.85(0.46,1.5)		64(55.2)	52(44.8)	0.9(0.46,1.8)	
Greater or equal to five	147(86.5)	23(13.5)	1.0		27(57.4)	20(42.6)	1.0	

*Place of delivery *								
Home	150(88.8)	19(11.2)	1.7(0.9,3.1)*∗∗*	1.5(0.7,3.1)	23(69.7)	10(30.3)	2.1(0.9,4.7)*∗∗*	2.4(0.9,5.8)
Health facility	136(82.4)	29(17.6)	1.0	1..0	68(52.3)	62(47.7)	1..0	1.0

*Sex of index child*								
Male	140(84.8)	25(15.2)	0.8(0.47,16)		56(62.2)	34(37.8)	1.7(0.9,3.3)*∗∗*	2.3(1.2,4.7)*∗*
Female	146(86.4)	23(13.6)	1.0		35(47.9)	38(52.1)	1.0	1.0

*Knowledge level*								
Poor	191(97.0)	6(3.0)	*14(5.7,34.3)∗*	13.4(5.3,34)*∗*	26(74.3)	9(25.7)	*2.8(1.2,6.4)∗*	2.7(1.3,6.5)*∗*
Good	95(69.3)	42(30.2)	1.0	1.0	65(50.8)	63(49.2)	1.0	1.0

*Heard about SSS?*								
No	221(89.2)	27(10.8)	*2.6(1.4,4.9)∗*	*1.3(0.6,2.8)*	54(64.3)	30(35.7)	*2.1(1.1,3.8)∗*	*1.6(0.8,3.4)*
Yes	65(75.6)	21(24.4)	1.0	1.0	37(46.8)	42(53.2)	1.0	1.0

*Have you Heard yogurt?*								
No	225(87.5)	32(12.5)	*1.8(0.9,3.5)∗∗*	1.1(0.5,2.3)	71(56.8)	54(43.2)	*1.2(0.57,2.4 )*	
Yes	61(79.2)	16(20.8)	1/0	1.0	20(52.6)	18(47.4)	1.0	

*Easy to get ORS?*								
No (Don't know)	74(84.1)	14(15.9)	*0.84(0.4,1.6)*		15(57.7)	12(42.3)	*1.1(0.47, 2.6)*	
Yes	212(86.2)	34(13.8)	1.0		76(55.5)	60(44.5)	1.0	

*Easy to prepare ORS*								
No (Don't know)	187(86.6)	29(13.4)	1.25(0.6,2.3)*∗∗*	2.4(1.3, 5.3)*∗*	23(79.3)	6(20.6)	3.7(1.4,9.7)*∗*	4.0(1.4,11.0)*∗*
Yes	98(83.8)	19(16.2)	1.0	1.0	68(50.7)	66(49.3)	1.0	1.0

*Easy to get Zinc?*								
No (Don't know)	246(89.5)	29(10.5)	*4.1(2.1, 8.0)∗*	2.4(1.2, 5.0)*∗*	57(56.4)	44(43.6)	1.07(0.57,2.02)	
Yes	40(67.8)	19(32.2)	1.0	1.0	34(54.8)	28(45.2)	1.0	

“*∗*”-indicates p-value less than 0.05, *∗∗* -Indicates P-value 0.05-0.25 in bi-variate analysis, “1.0” indicates reference group. SSS^∧^- indicates “salt-sugar solution”, ORS^∧^ indicates “Oral rehydration solution, *∗∗∗=can't read and write and informal education,∗∗∗∗= primary, secondary, tertiary*,* others∗∗∗∗∗*- merchant and daily laborer

## Data Availability

The data used to support the findings of this study are available from the corresponding author upon request.
